# A lipid droplet-associated protein Nem1 regulates appressorium function for infection of *Magnaporthe oryzae*

**DOI:** 10.1007/s42994-023-00098-5

**Published:** 2023-02-18

**Authors:** Deng Chen, Xuan Cai, Junjie Xing, Shen Chen, Juan Zhao, Zhiguang Qu, Guotian Li, Hao Liu, Lu Zheng, Junbin Huang, Xiao-Lin Chen

**Affiliations:** 1 State Key Laboratory of Agricultural Microbiology and Provincial Key Laboratory of Plant Pathology of Hubei Province, College of Plant Science and Technology, Huazhong Agricultural University, Wuhan, 430070 China; 2 State Key Laboratory of Hybrid Rice, Hunan Hybrid Rice Research Center, Changsha, 410125 China; 3 Guangdong Provincial Key Laboratory of High Technology for Plant Protection, Plant Protection Research Institute, Guangdong Academy of Agricultural Sciences, Guangzhou, 510640 China

**Keywords:** Lipid biogenesis, TOR signaling, cAMP-PKA signaling, Conidium formation, Appressorium, *Magnaporthe oryzae*

## Abstract

Lipid droplets are important storages in fungal conidia and can be used by plant pathogenic fungi for infection. However, the regulatory mechanism of lipid droplets formation and the utilization during fungal development and infection are largely unknown. Here, in *Magnaporthe oryzae*, we identified a lipid droplet-associated protein Nem1 that played a key role in lipid droplets biogenesis and utilization. Nem1 was highly expressed in conidia, but lowly expressed in appressoria, and its encoded protein was localized to lipid droplets. Deletion of *NEM1* resulted in reduced numbers of lipid droplets and decreased content of diacylglycerol (DAG) or triacylglycerol (TAG). *NEM1* was required for asexual development especially conidia production. The Δ*nem1* mutant was nearly loss of virulence to host plants due to defects in appressorial penetration and invasive growth. Remarkably, Nem1 was regulated by the TOR signaling pathway and involved in the autophagy process. The Ser303 residue of Nem1 could be phosphorylated by the cAMP-PKA signaling pathway and was important for biological function of Nem1. Together, our study revealed a regulatory mechanism of lipid biogenesis and metabolism during the conidium and appressorium formation of the rice blast fungus.

**Supplementary Information:**

The online version contains supplementary material available at 10.1007/s42994-023-00098-5.

## Introduction

Most eukaryotic cell organelles are enclosed by a membrane consisted of proteins and a lipid bilayer (Pillai et al. 2017). The major components of the lipid bilayer are phospholipids which play a crucial role in membrane biogenesis and lipid metabolism (Eastmond et al. 2010; Nakamura et al. 2009; Pillai et al. 2018). Cytoplasmic lipid droplets (LDs), existing in almost all eukaryotic cells, are important organelles responsible for storing and providing lipid to meet required energy (Graef 2018; Henne et al. 2019). LDs are often considered originally form the endoplasmic reticulum (ER) membrane and are evolutionarily conserved. The main forms of lipids stored in LDs are neutral lipids containing triacylglycerols (TAGs) and/or sterol esters (SEs). Phosphatidate (PA) is the key precursor of lipid metabolism (Henne et al. 2019; Karanasios et al. 2013). On the one hand, dephosphorylation of PA produces diacylglycerol (DAG) which can be acylated to TAG to be stored in rich nutrients. On the other hand, PA can be converted to cytidine diphosphate diacylglycerol (CDP-DAG), which forms membrane phospholipids including phosphatidylserine (PS), phosphatidylethanolamine (PE), and phosphatidylcholine (PC) to promote cell growth and proliferation (Barbosa et al. 2015; Dubots et al. 2014; Karanasios et al. 2013). Because phospholipids and TAG share the same precursor, it is necessary for cells to coordinate the two pathways in which many important proteins are involved.

Lipins, large proteins mainly found in the cytosol, are Mg^2+^-dependent phosphatidate phosphatases, which catalyzes the conversion from PA to DAG by promoting dephosphorylation of PA (Santos-Rosa et al. 2005). In mammals, three *LIPIN* genes, *LIPIN1*-*3*, were identified to have different but overlapping expression patterns. Disrupting lipins genes led to lipid metabolic disorders, rhabdomyolysis, peripheral neuropathy, and inflammation (Chen et al. 2015; Santos-Rosa et al. 2005). In *Caenorhabditis elegans*, low lipin expression affected the dynamics of the peripheral ER and nuclear envelope (Jung et al. 2020). In *Saccharomyces cerevisiae*, a single lipin orthologue named *PAH1* was found. Loss of *PAH1* in yeast led to slow growth, abnormal expansion of nuclear/ER membrane, and disordered lipid metabolism (Fang et al. 2014; Hsu et al. 2021; Kwiatek et al. 2020). Activated *PAH1* required to be dephosphorylated by the nuclear/ER membrane-associated protein phosphatase complex consisting of Nem1 (catalytic subunit) and Spo7 (regulatory subunit) (Liu et al. 2019). The two subunit proteins both possess two transmembrane-spanning domains. Nem1 binds to Spo7 through its conserved C-terminal domain, and this association is responsible for the formation of the complex in the membrane bilayer (Siniossoglou et al. 1998). Nem1 is a member of the haloacid dehalogenase superfamily, and its phosphatase activity depends on the DXDX(T/V) catalytic motif within its HAD-like domain. Spo7, which binds to the catalytic domain of Nem1, is essential for the activity of the phosphatase complex (Dubots et al. 2014; Kim et al. 2007). Nem1-Spo7-mediated dephosphorylation of Pah1 activates the catalytic efficiency of Pah1, but meanwhile primes it for proteasome-dependent degradation. Therefore, dephosphorylated Pah1 is both active and unstable, which is likely to be a constraint step preventing excess PA into the synthesis of TAG (Bahmanyar 2015; Karanasios et al. 2013; Zhang et al. 2018). The function of the phosphatase Nem1-Spo7 axis is important, but the underlined upstream regulatory mechanism is largely unknown.

*Magnaporthe oryzae*, the causal agent of rice blast, leads to severe yield loss of rice every year (Dean et al. 2012; Ebbole 2007). Infection of this ascomycete starts from adhesion of conidia on the host plants. In proper environment, conidia geminate to form a specialized structure known as appressorium. The appressorium accumulates enormous turgor pressure to form a penetration peg to rupture plant cuticles. Inside the host cell, the primary invasive hypha will differentiate to bulbous biotrophic invasive hypha for expansion and blast lesions formed with numerous conidia at the late necrotrophic stage (Fernandez et al. 2018; Martin-Urdiroz et al. 2016; Wilson et al. 2009).

The appressorium formation and maturation is a key process required for successful infection. Several signaling pathways have been reported to play vital roles in the appressorium formation, including cAMP-PKA, Pmk1-MAPK, and TOR signaling pathways (Wilson et al. 2009). During the appressorium formation and maturation, a series of cellular processes, such as utilization of lipids, are activated (Chen et al. 2017; Eseola et al. 2021; Kubo 2013; Yan et al. 2016). However, the underlined mechanism linked lipid biogenesis in conidium and the utilization in appressorium remain to be revealed. In this study, a *M. oryzae* lipid droplet protein Nem1 was identified and found to be involved in lipid droplets formation during conidia formation and lipid utilization during appressoria formation. Importantly, we found that Nem1 was by both the TOR signaling pathway and the cAMP-PKA signaling pathway. Further, a phosphorylation site of Nem1 at Ser303 found to be regulated by cAMP-PKA signaling pathway and necessary for full function of Nem1. Our results revealed a novel regulatory mechanism of lipid droplets during the appressorium formation of *M. oryzae*.

## Results

### Identification of NEM1

**Fig. 1:**
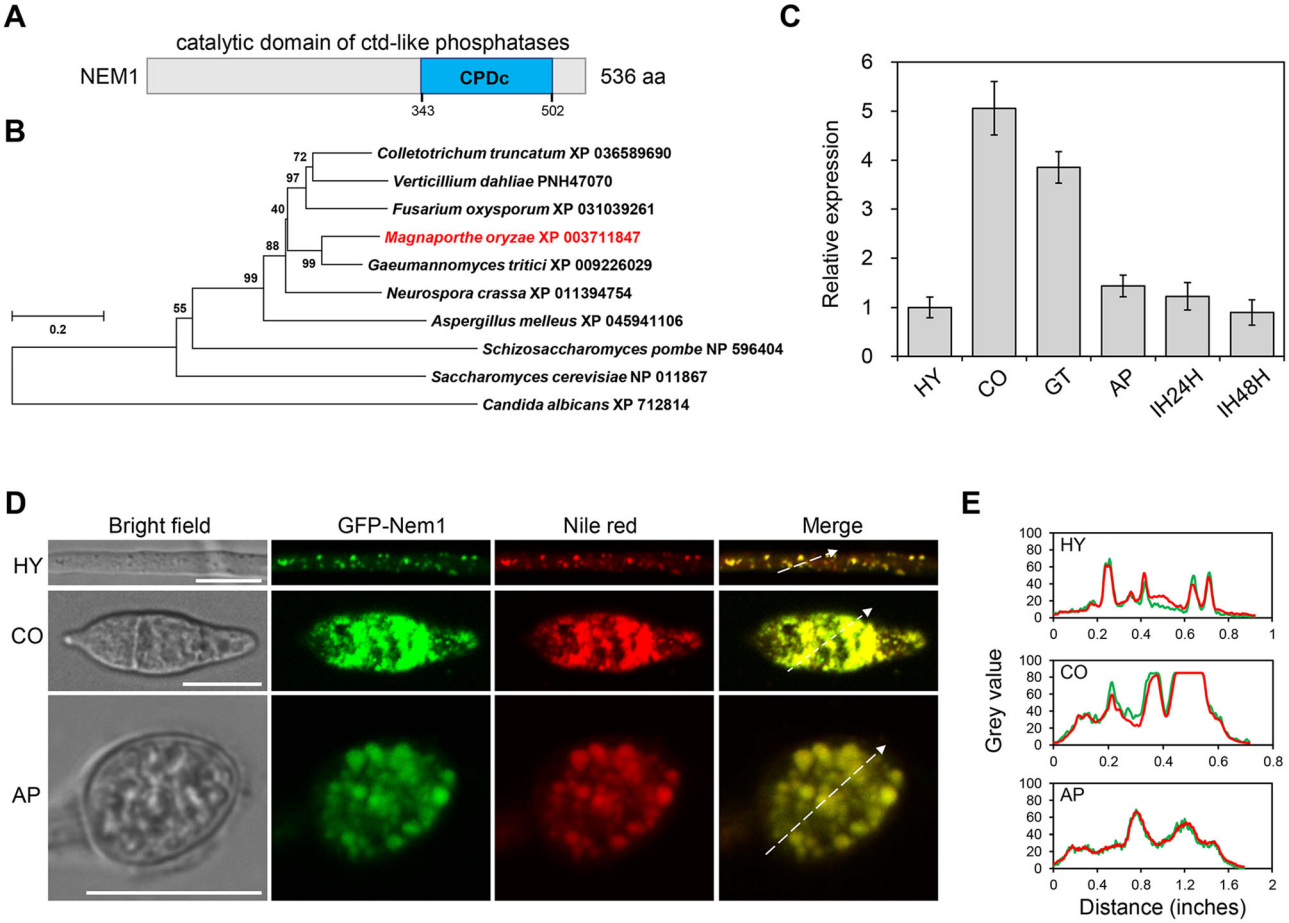
Protein property of Nem1. **A** Protein domain analysis of Nem1. CPDc, catalytic domain of ctd-like phosphatases. **B** Evolutional analysis of Nem1 among fungi. Nem1 of *M. oryzae* was labelled in red. **C** Relative gene expression of *NEM1* at different developmental stages. HY, hypha; CO, conidium; GT, germ tube; AP, appressorium at 12 hpi; IH24H, invasive hypha at 24 hpi; IH48H, invasive hypha at 48 hpi. **D** Nem1 is localized to the lipid droplets in the hypha and conidium. The transformant expressing Nem1-GFP was stained with Nile red and observed under a confocal microscope. Scale bar: 10 μm. HY, hypha; CO, conidium; AP, appressorium. E Line-scan graph analysis showed that Nem1-GFP was co-localized with Nile red-stained lipid droplets

In *S. cerevisiae*, a protein named Nem1 (nuclear envelope morphology protein 1) is found to be required for Pah1 dephosphorylation or activation and is essential for lipid metabolism and TAG synthesis (Bahmanyar 2015; Karanasios et al. 2013; Liu et al. 2019; Zhang et al. 2018). Through a BLAST search, we identified a homologous protein of Nem1 in *M. oryzae*, MGG_06001. *M. oryzae* Nem1 contains 536 amino acids with a long CPDc (catalytic domain of ctd-like phosphatases) domain near its C-terminus (Fig. 1A). Phylogenetic analysis shows that Nem1 is highly conserved among fungi especially the plant pathogenic fungi, such as *Fusarium oxysporum*, *Gaeumannomyces tritici*, and *Verticillium dahliae* (Fig. 1B).

To investigate function of Nem1 in *M. oryzae*, we deleted *NEM1* in the wild-type strain P131 using a split-marker approach (Fig. S1A). Two independent deletion mutants, NEM1KO1 and NEM1KO2, were gained and confirmed by a PCR-mediated verification (Fig. S1B). We also obtained the complementary strains by introducing the vector pKN-NEM1, which contains the coding region of *NEM1* driven by its native promoter, to NEM1KO1 (Fig. S2A). All transformants have been verified by PCR amplification of the *NEM1* gene, and their phenotypes were all similar to P131 (Fig. S2B-E). One of them, cNEM1, was chosen for further analysis.

To investigate the possible biological function in *M. oryzae*, the expression profile of *NEM1* was detected at different developmental and infection stages, including vegetative hyphae (HY), conidia (CO), germ tubes (GT), appressoria (AP), the early (IH18H) and late (IH48H) stage of invasive hyphae. *NEM1* was highly expressed at conidia and germ tube (immature appressorium) stages (Fig. 1C), suggesting that NEM1 may play roles in conidia formation and appressoria formation.

### NEM1 is localized in lipid droplets

To further study the molecular function of NEM1, subcellular localization observation was conducted in *M. oryzae*. Since Nem1 is predicted to be involved in lipid synthesis, we hypothesized that *M. oryzae* Nem1 is localized to the lipid droplet, an organelle responsible for lipid storage. The Nem1 protein driven by its native promoter fused with the green fluorescent protein (GFP) was expressed in P131. Transformants expressing Nem1-GFP were selected to observe fluorescent signals. The lipophilic dye Nile red was used as a control to stain lipid droplets in cells of the transformant strain. Strong punctate Nem1-GFP signal was well co-localized with Nile red-stained lipid droplets in vegetative hyphae, conidia, and appressoria (Fig. 1D). The two fluorescence signals were well overlapped with the line-scan graph analysis (Fig. 1E). Therefore, we concluded that Nem1 is a lipid droplet localized protein.

### Nem1 is required for lipid accumulation in mycelium and conidium

**Fig. 2:**
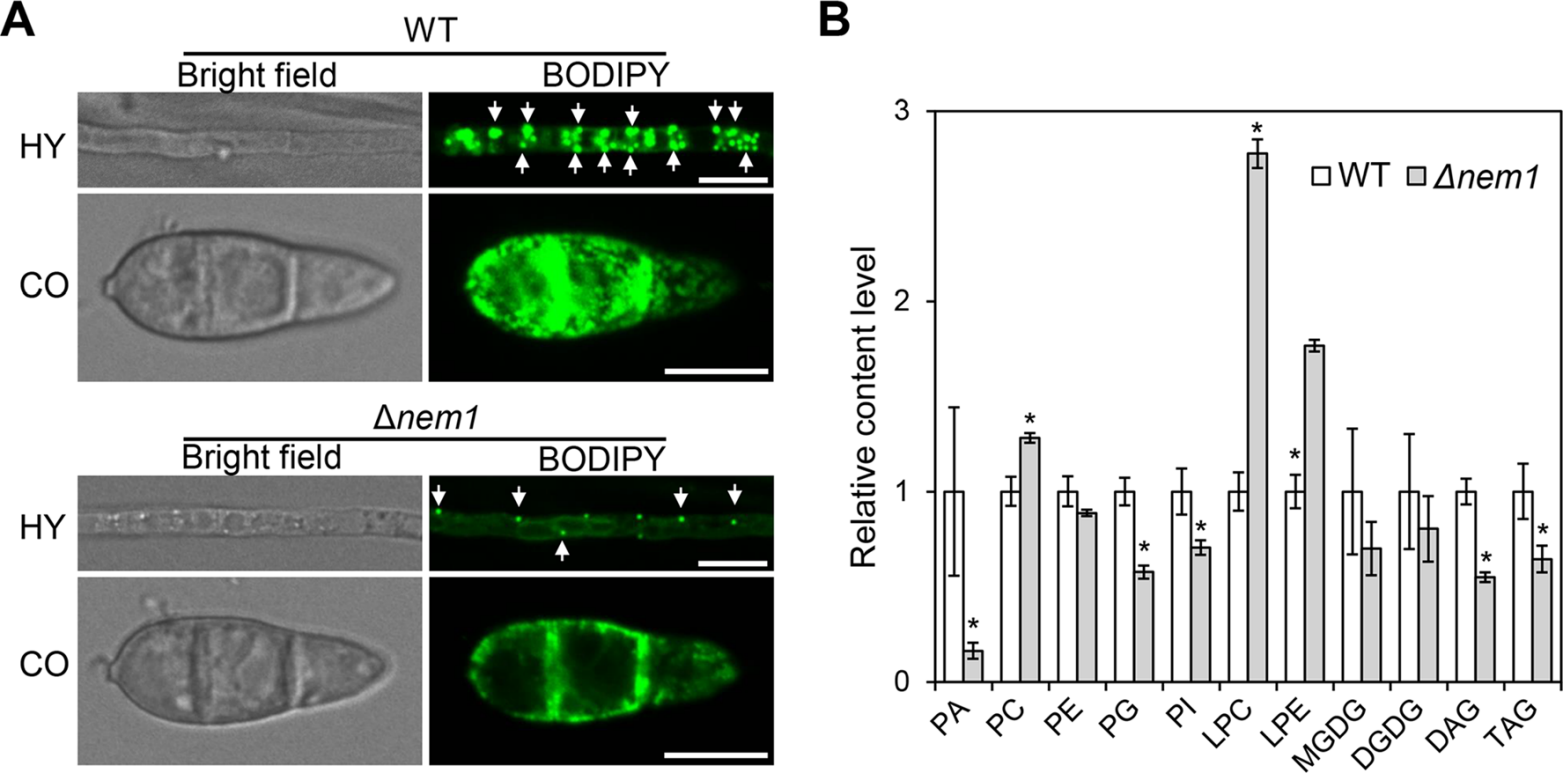
Nem1 is important for lipid accumulation and composition.** A** Lipid droplets were stained by BODIPY 493/503 in hyphae (HY) and conidia (CO) of WT and Δ*nem1*. White arrows indicate lipid droplets strained by BODIPY 493/503. Scale bars: 10 μm. **B** Relative content of lipid composition in WT and Δ*nem1* by lipidomics analysis. Asterisks indicate statistically significant differences (*P* < 0.05). PA, phosphatidic acid; PC, phosphatidylcholine; PE, phosphatidylethanolamine; PG, phosphatidyl glycerol; PS, phosphatidylserine; PI, phosphatidylinositol; MGDG, monogalactosyl diglyceride; DGDG, digalactosyl diglyceride; LPC, lysophosphatidyl choline; LPE, lysophosphatidyl ethanolamine; DAG, diacylglycerol; TAG, triacylglycerol

Because Nem1 is localized to lipid droplets, we then investigated whether Nem1 is required for lipid biogenesis. BODIPY 493/503, a lipophilic fluorescent probe, was used to label lipid droplets in mycelium and conidium. A great number of lipid droplets were observed in mycelium of P131, while far fewer lipid droplets were detected in the Δ*nem1*. In the conidium, numerous lipid droplets were observed in P131, far more than that of Δ*nem1* (Fig. 2A). These results demonstrated that lipid content was significantly reduced in Δ*nem1* compared with P131. Further, thin-layer chromatography (TLC) assay was conducted to detect lipid contents between P131 and Δ*nem1*. Lipid composition including PA (phosphatidic acid), PC (phosphatidylcholine), PE (phosphatidylethanolamine), PS (phosphatidylserine), PI (phosphatidylinositol), PG (phosphatidyl glycerol), CL (cardiolipin), DGDG (digalactosyl diglyceride), and SQDG (sulphoquinovosyl diglyceride) was segregated, and several compositions (PA, PC, PS, PE, PG, and PI) showed difference in content indicated by spot areas (Fig. S3). Lipidomics analysis demonstrated that the content of PA, PG, and PI were all significantly reduced in Δ*nem1* compared with that in P131. Meanwhile, the level of PC, LPC (lysophosphatidyl choline), and LPE (lysophosphatidyl ethanolamine) in Δ*nem1* was obviously higher than that in P131. More importantly, the relative contents of DAG (diacylglycerol) and TAG (triacylglycerol) were both significantly reduced in Δ*nem1* (Fig. 2B). These results indicate that Nem1 affects accumulation and composition of lipids in *M. oryzae*.

### Nem1 is important for asexual development of *M. oryzae*

**Fig. 3:**
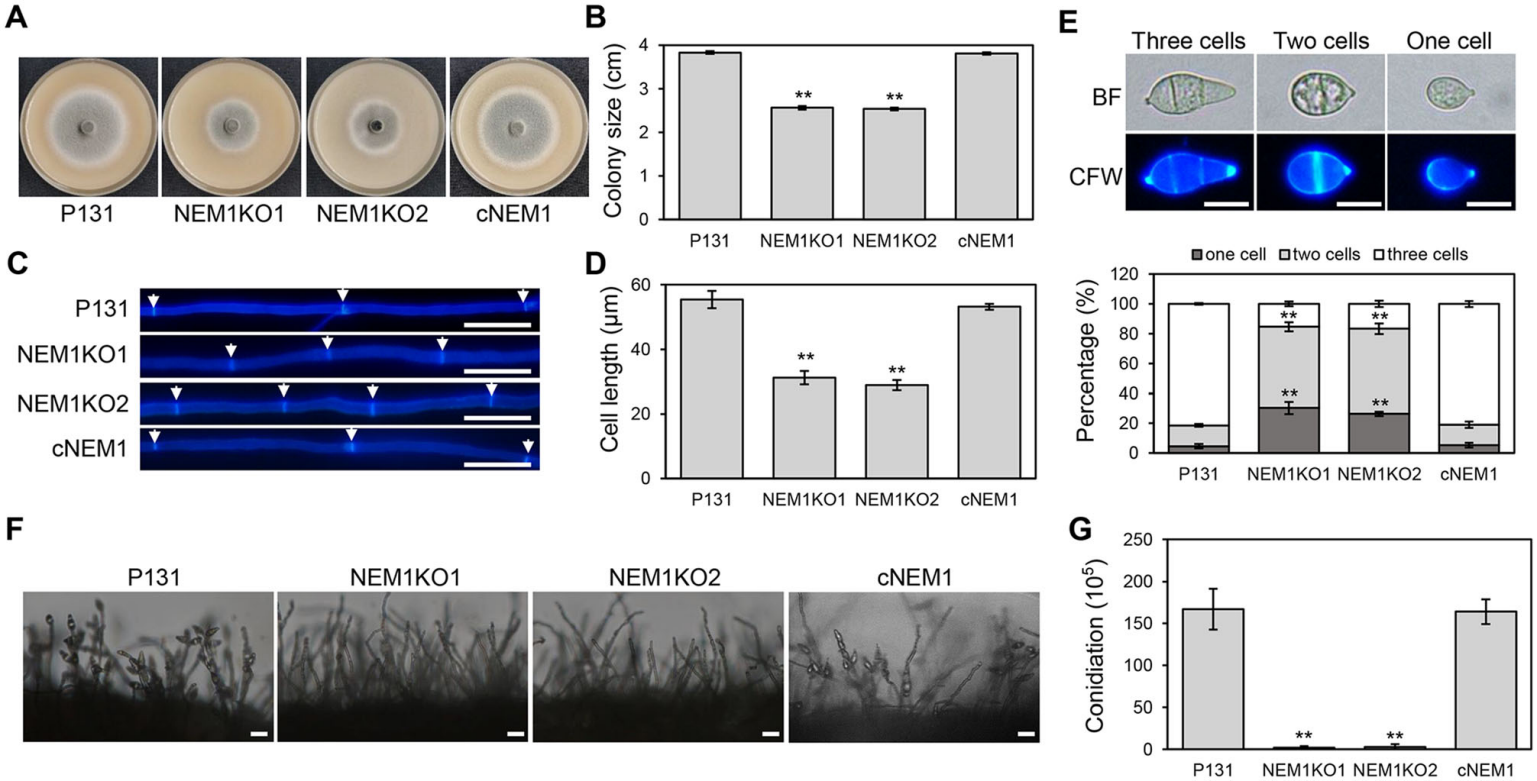
Nem1 is necessary for asexual development of *M. oryzae*. **A** Colonies of P131, NEM1KO1, NEM1KO2, and cNEM1 grew on OTA plate for 5 days. **B** Statistical analysis of the colony diameter of the indicated strains. Asterisks indicate statistically significant differences (*P* < 0.01). **C** Hyphal tip of P131, NEM1KO1, NEM1KO2, and cNEM1 were stained by CFW. White arrows show septa of hyphae. Scale bar: 20 μm. **D** Statistical analysis of near apical hyphal tip cell length of the indicated strains. Asterisks indicate statistically significant differences (*P* < 0.01). **E** Statistical analysis of percentages of conidia with three cells, two cells, and one cell. Asterisks indicate statistically significant differences (*P* < 0.01). Scale bar: 10 μm. **F** Conidiophores of P131, NEM1KO1, NEM1KO2, and cNEM1 were observed under the microscope. Scale bar: 20 μm. **G** Statistical analysis of conidiation of the indicated strains. P131, the wild-type strain; NEM1KO1 and NEM1KO2, deletion mutants of *NEM1*; cNEM1, the NEM1 complementary strain. Asterisks indicate statistically significant differences (*P* < 0.01)

Because Nem1 is required for lipid biogenesis of the mycelium and conidium, we then tested if it affects asexual development of *M. oryzae*. When grown on oatmeal agar (OTA) plates for 5 days, the colony diameter of NEM1KO1 or NEM1KO2 was around 2.5 cm, far smaller than that of P131 or cNEM1 (~ 4.0 cm) (Fig. 3A, B). Moreover, the cell wall and septa of the hyphal tips were stained with CFW to determine the hyphal cell length. The average length of P131 or cNEM1 hyphae was significantly longer than those of NEM1KO1 or NEM1KO2 (Fig. 3C, D). Cell numbers of conidium were also determine using CFW to stain the conidial septa. Around 80% of P131 or cNEM1 conidia had three cells, whereas the percentage of NEM1KO1 or NEM1KO2 was only 20%. The ratios of conidia with two cells and one cell were much higher than that of the wild-type or complementary strains (Fig. 3E). Further, conidia production was detected among these strains. Numerous conidia were formed on conidiophores of P131 or cNEM1, but sparse conidia were observed on conidiophores of the deletion mutants (Fig. 3F). Consistent with this, the conidiation of the Δ*nem1* mutants (approximately 2.5 × 10^5^/mL per 6-cm-diameter plate) was only about 2% of P131 or cNEM1 (approximately 1.7 × 10^7^/mL per 6-cm-diameter plates) (Fig. 3G). Taken together, our results demonstrated that Nem1 is important for vegetative growth, conidial morphology, and conidia production.

### Nem1 is required for virulence of *M. oryzae*

**Fig. 4:**
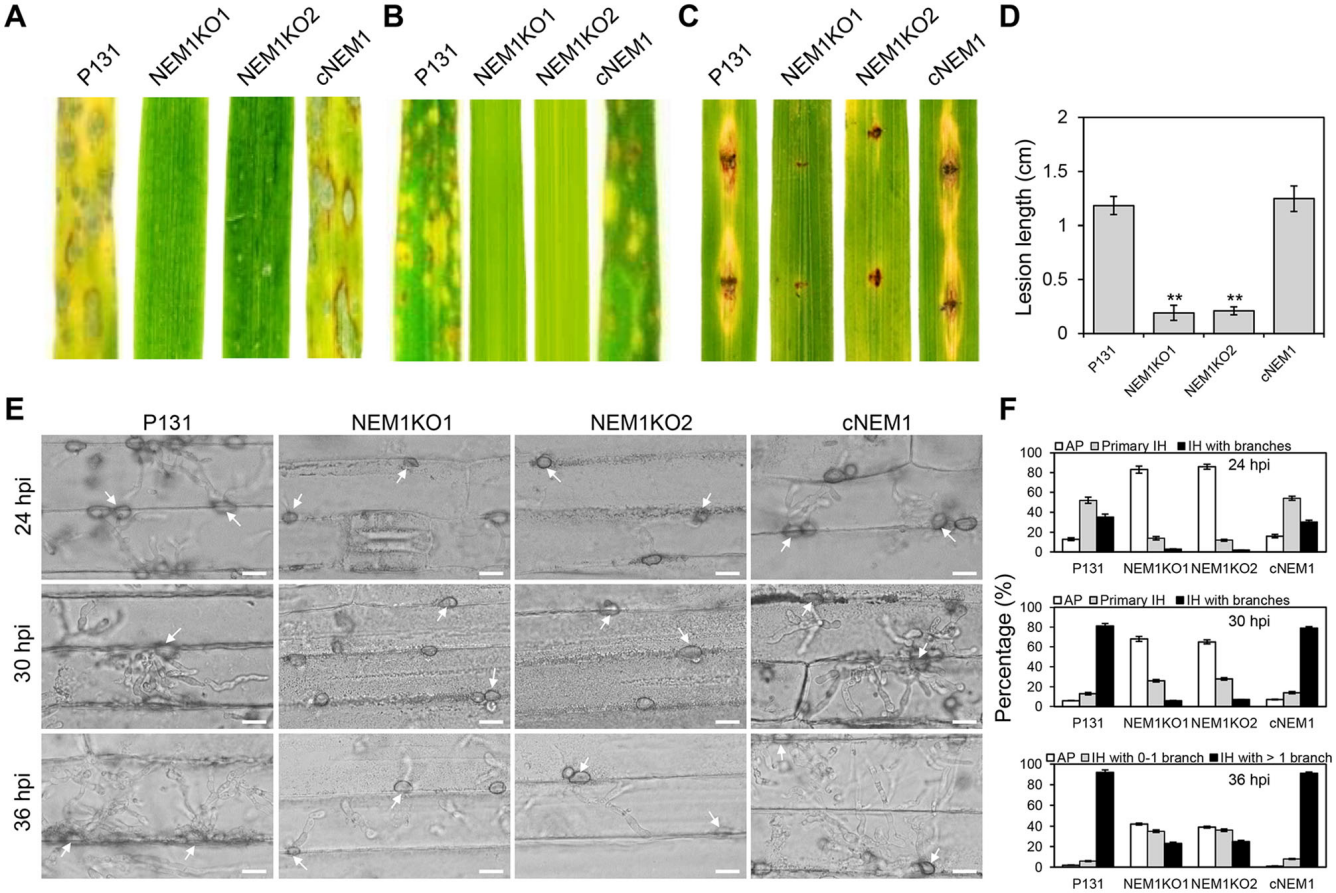
Nem1 is required for full virulence of *M. oryzae*. **A** Barley leaves were inoculated by spraying with conidial suspension of P131, NEM1KO1, NEM1KO2, and cNEM1. **B** Rice seedlings were inoculated by spraying with conidial suspension of the indicated strains. **C** Scratched rice leaves were inoculated by conidial suspension of the indicated strains. **D** Statistical analysis of length of lesion produced from wounded sites in **(C)**. Asterisks indicate statistically significant differences (*P* < 0.01). **E** Invasive growth of P131, NEM1KO1, NEM1KO2, and cNEM1 at different time points were observed under a microscope. White arrows indicate appressoria. Scale bar: 10 μm. **F** Statistical analysis of percentages of AP and branched IH in **(E)**. AP, appressorium; IH, invasive hypha

To investigate the role of Nem1 in pathogenicity, conidial suspension of P131, NEM1KO1, NEM1KO2, and cNEM1 were spray-inoculated on barley leaves and rice seedlings. Numerous lesions were observed on leaves infected with P131 or cNEM1, whereas lesions could hardly produce on leaves inoculated with the Δ*nem1* mutants (Fig. 4A, B). Further, inoculations on scratched rice leaves were also performed by mycelial blocks. Compared with the large-sized lesions caused by P131 or cNEM1, almost no expanded lesions were found on rice leaves inoculated with NEM1KO1 or NEM1KO2 (Fig. 4C, D). To further determine which infectious stage was affected in the mutants, the infection process was observed on barley epidermis inoculated with different strains. At 24 h post-inoculation (hpi), the majority of P131 or cNEM1 appressoria had formed invasive hyphae (IH). However, the Δ*nem1* mutants still maintained at the appressoria stage. Later at 30 hpi, the percentage of IH caused by P131 or cNEM1 reached more than 90%, while it was no more than 40% caused by the Δ*nem1* mutants. At 36 hpi, there were still around 80% appressoria that were unable to form branched IH, while the majority of P131 or cNEM1 had formed IH with more than one branch (Fig. 4E, F). In conclusion, Nem1 is required for virulence and invasive growth of *M. oryzae*.

### Nem1 is required for the appressorium formation and appressorial lipid droplets utilization

**Fig. 5:**
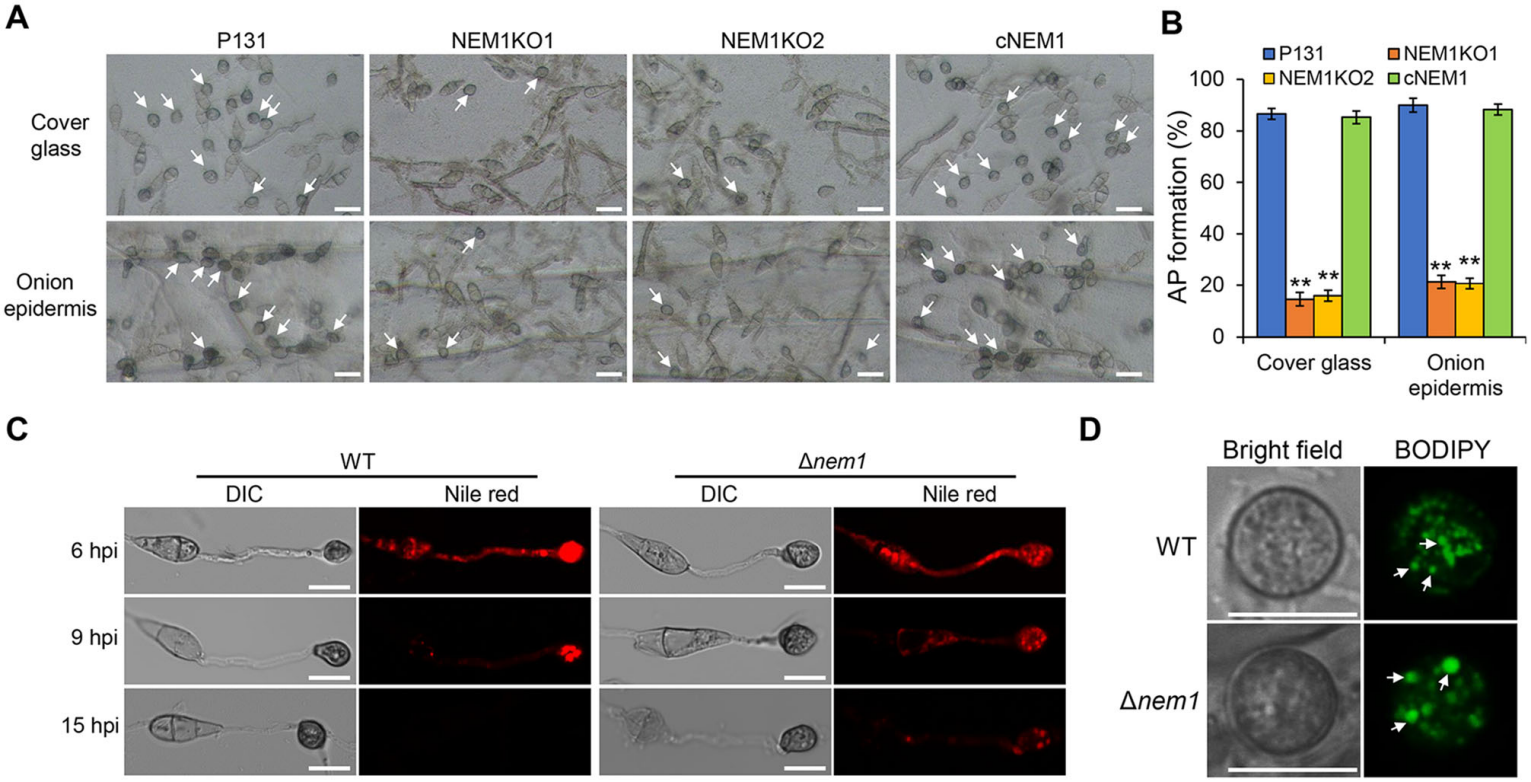
Nem1 is required for appressoria formation. **A** Observation of appressoria formation of P131, NEM1KO1, NEM1KO2, and cNEM1 on the cover glass. White arrows indicate appressoria. **B** Statistical analysis of the AP formation rate in **(A)**. Asterisks indicate statistically significant differences (*P* < 0.01). **C** Lipid turnover during appressoria formation of P131 and Δ*nem1* was stained with Nile red at different time points. Scale bar: 10 μm. **D** Lipid droplets were stained by BODIPY 493/503 in the appressorium of WT and Δ*nem1* at 10 hpi. White arrows indicate the strained lipid droplets. Scale bar: 10 μm

As lipid turnover is necessary for appressoria formation of *M. oryzae*, we then tested whether appressoria formation was affected in the Δ*nem1* mutants. Conidial suspension of different strains was dropped on the hydrophobic surface. At 24 hpi, the appressoria formation percentages of P131 and cNEM1 were nearly 90%, while it was less than 20% of the Δ*nem1* mutants. On onion epidermis, the tendency of AP formation was in consistence with that on the hydrophobic surface (Fig. 5A, B). These results showed that Nem1 is essential for appressoria formation.

We also intended to detect whether appressorial turgor of the Δ*nem1* mutant was affected, however, it was hard for us to perform the cytorrhysis assay. However, we were able to detect if utilization of lipid droplets was affected. On the hydrophobic surface, the lipid droplets in the wild-type strain were quickly utilized and degraded during the process of appressoria formation, and gradually disappeared at 15 hpi. While in the Δ*nem1* mutant, lipid droplets were still observable at 15 hpi (Fig. 5C). We also noticed that at 10 hpi, lipid droplets in WT appressoria were much smaller than that in the Δ*nem1* mutant (Fig. 5D), further indicating that the lipid droplets degradation process was affected in the mutant. These results demonstrate that Nem1 is required for lipid droplets utilization during the appressorium maturation, which may affect appressorial turgor accumulation and penetration.

### Nem1 is important for cell wall integrity and stress response

To determine if Nem1 plays roles in stress response, cell wall integrity perturbing agents (Calcofluor white [CFW], Congo red [CR], and sodium dodecyl sulfate [SDS]), and osmotic pressure agents (0.7 M NaCl and 1.0 M Sorbitol) were separately added to the complete medium (CM) plates for testing. The Δ*nem1* mutant was more sensitive to 0.1 mg/mL CFW or 0.2 mg/mL CR, but not 0.005% SDS. The Δ*nem1* mutant was slightly sensitive to 0.7 M NaCl, while it was more resistant to 1.0 M Sorbitol. This result shows Nem1 is involved in cell wall integrity and osmotic pressure response (Fig. S4A, B).

Since the *NEM1* deletion mutants are defective in invasive growth, we speculated during infection, the mutants encountered inhibition from host reactive oxygen species (ROS). As expected, compared with WT, the Δ*nem1* mutant was more sensitive to different concentrations of H_2_O_2_ (10 mM, 15 mM, or 20 mM), suggesting Nem1 plays a role in ROS response (Fig. S4C, D). Cellular ROS produced from host plants infected by P131, Δ*nem1*, and cNEM1 at 30 hpi was detected with 3,3'-diaminobenzidine (DAB). Compared with P131 or cNEM1, ROS accumulation was more often observed in barley epidermal cells infected with Δ*nem1* (Fig. S2E, F). Therefore, limited invasive growth of Δ*nem1* might be caused by defect in scavenging host ROS. To confirm this, diphenyleneiodonium (DPI) was used to inhibit the host NADPH oxidase activity, which is necessary for host ROS generation (Liu et al. 2021). Compared with the DMSO treatment control, 0.5 μM DPI treatment resulted in a partial recovery of the invasive growth of Δ*nem1* (Fig. S2G). These results suggest that Nem1 plays an important role in stress response and scavenging host ROS.

### Nem1 regulates autophagy process

**Fig. 6:**
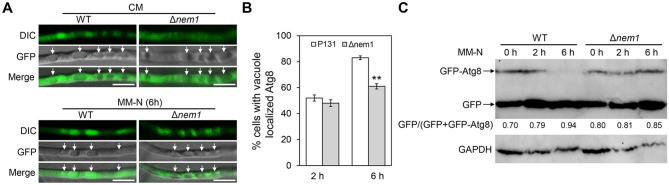
Nem1 regulates autophagy in *M. oryzae*. **A** Observation of GFP-Atg8 localization in WT and Δ*nem1* cultured in CM for 48 h followed by MM-N for 0 h and 6 h. White arrows indicate vacuoles. Scale bar: 20 μm. **B** Statistical analysis of percentage of cells with vacuole localized Atg8. Asterisks indicate statistically significant differences (*P* < 0.01). **C** Western blotting analysis of GFP-Atg8 and free GFP in the transformant WT/ GFP-Atg8 and Δ*nem1*/ GFP-Atg8 cultured in MM-N for 2 h and 6 h. The numerical value showing gray value ratio of free GFP to the sum of free GFP and fused GFP-Atg8. GAPDH was loaded as a control

Since Nem1 is required for appressorium maturation, we wondered if Nem1 regulates autophagy in *M. oryzae*. A GFP-Atg8 fusion construct was respectively transformed into WT and Δ*nem1*. Resulting transformants confirmed by Western blotting were cultured in minimum medium without nitrogen (MM-N). When cultured in MM-N for 6 h, compared with that in wild-type cells, less GFP-Atg8 was localized to the vacuole in Δ*nem1* cells (Fig. 6A, B). This result indicated the autophagy process was affected in Δ*nem1*. Total protein levels of GFP-Atg8 in WT or Δ*nem1* cultured in MM-N for 2 h and 6 h were also quantified to determine autophagy level by immunoblot. The extent of autophagy was estimated by the intensity ratio of free GFP to the intact GFP-Atg8 and free GFP together (GFP/[GFP + GFP-Atg8]). When the WT mycelium was treated with MM-N, the ratio remarkably increases from 0.7 (0 h) to 0.79 (2 h) and 0.94 (6 h). By contrast, in Δ*nem1*, the value (GFP/[GFP + GFP-Atg8]) slightly increased from 0.8 (0 h) to 0.85 (6 h) (Fig. 6C). This result means autophagy is blocked in the Δ*nem1* mutant, indicating the autophagy process of *M. oryzae* is regulated by Nem1.

### Nem1 is involved in the TOR signaling pathway

**Fig. 7:**
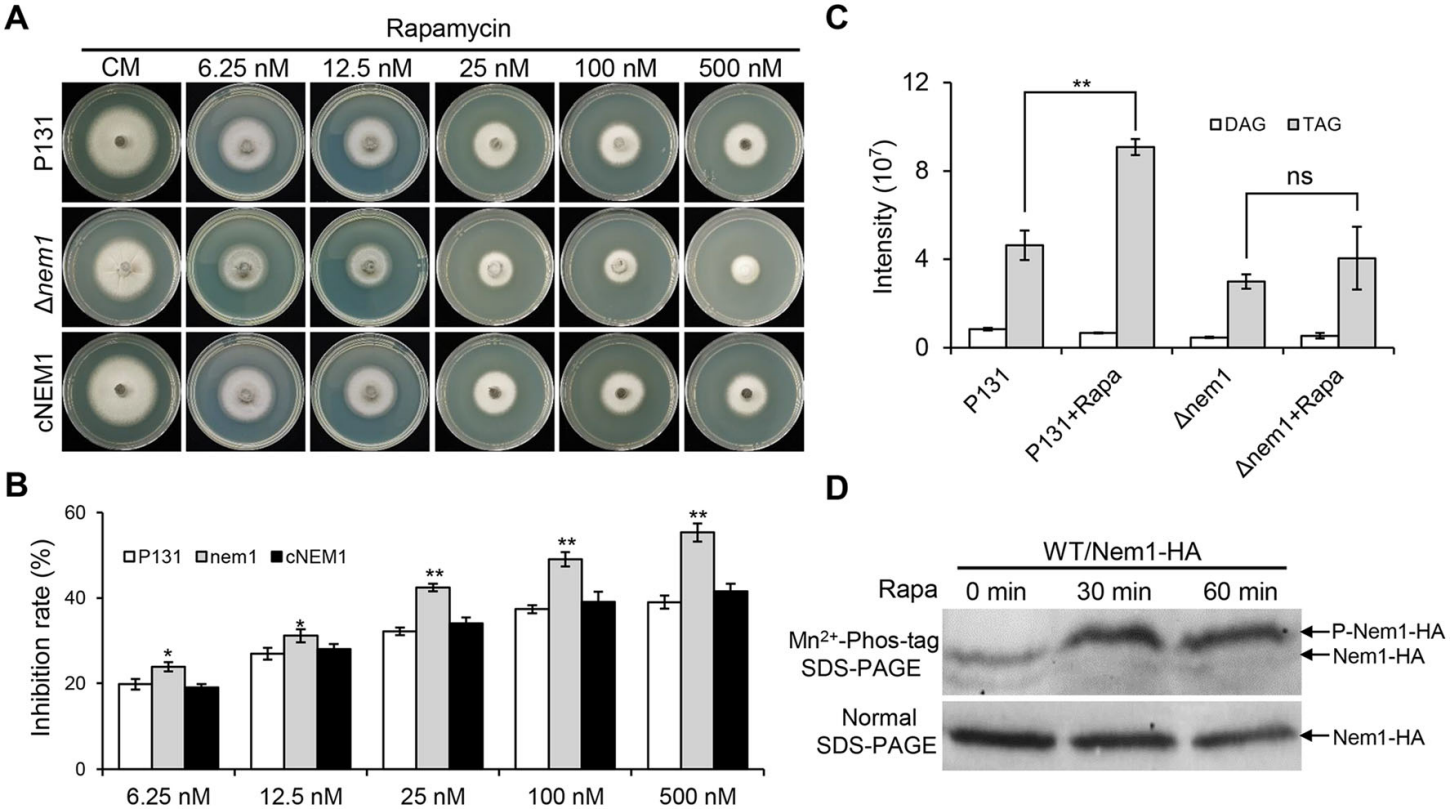
Nem1 is regulated by the TOR signaling pathway. **A** P131, Δ*nem1*, and cNEM1 were cultured on CM plates added with 6.25 nM, 12.5 nM, 25 nM, 100 nM, or 500 nM rapamycin for 5 days. **B** Statistical analysis of growth inhibition rate of the indicated concentration of rapamycin. Asterisks indicate statistically significant differences (*P* < 0.05). **C** Statistical analysis of relative DAG and TAG content of P131 and Δ*nem1* with or without rapamycin induction. Asterisks indicate statistically significant differences (*P* < 0.01). ns, no significance.** D** Cell extracts of transformants expressed Nem1-HA in WT induced with rapamycin for 30 min and 60 min were subjected to Phos-tag SDS-PAGE analysis. The normal SDS-PAGE was used as a control

Because the TOR signaling is important for the appressorium formation which is strongly affected by Nem1, we then tested whether the Δ*nem1* mutant was sensitive to rapamycin, which can inactivate the TOR complex 1 (TORC1). Gradient concentrations of rapamycin from 6.25 nM to 500 nM were added in the CM plates to detect colony diameter of different strains. As expected, compared to that of P131 and cNEM1, the Δ*nem1* was more sensitive to rapamycin in every concentration (Fig. 7A, B). Since Nem1 is a protein related to lipid metabolism, we hypothesized that DAG and TAG contents may be affected by rapamycin. To test this hypothesis, total lipid in the mycelium of P131 and Δ*nem1* treated with 25 nM rapamycin was extracted for lipidomics analysis. The result of lipidomics showed that the TAG content of P131 treated by rapamycin was almost two-fold of the untreated one. By contrast, no significant difference of TAG content of Δ*nem1* was detected between rapamycin treated and untreated conditions. Relative DAG content of the untreated P131 or Δ*nem1* was considerable to that treated by rapamycin (Fig. 7C). This result indicated that Nem1-mediated TAG synthesis is affected by the TOR signaling pathway.

In budding yeast, Pah1 phosphatidate phosphatase activity is regulated by TORC1. In parallel, Pah1 activation requires the Nem1-Spo7 phosphatase complex which in turn is often phosphorylated in the cell (Dubots et al. 2014; Su et al. 2018). We wondered if phosphorylation of Nem1 was affected by TORC1 in *M. oryzae*. To verify this assumption, the Nem1-HA fusion construct was transformed into WT. The resulting strain WT/Nem1-HA was cultured in CM treated with 20 nM rapamycin for 30 min or 60 min. Total proteins were extracted and subjected to be analyzed on gels containing the Phos-tag, which retards the mobility of phosphoproteins. As shown in Fig. 7D, Western blotting using anti-HA as an antibody showed the mobility shift of the WT/Nem1-HA. The shift of Nem1-HA treated with rapamycin for 30 min or 60 min was slower than that of the untreated one, suggesting a phosphorylation of Nem1 (Fig. 7D). Together, phosphorylation of Nem1 is regulated by the TOR signaling pathway in *M. oryzae*.

### Nem1 is regulated by cAMP-PKA signaling pathway

**Fig. 8:**
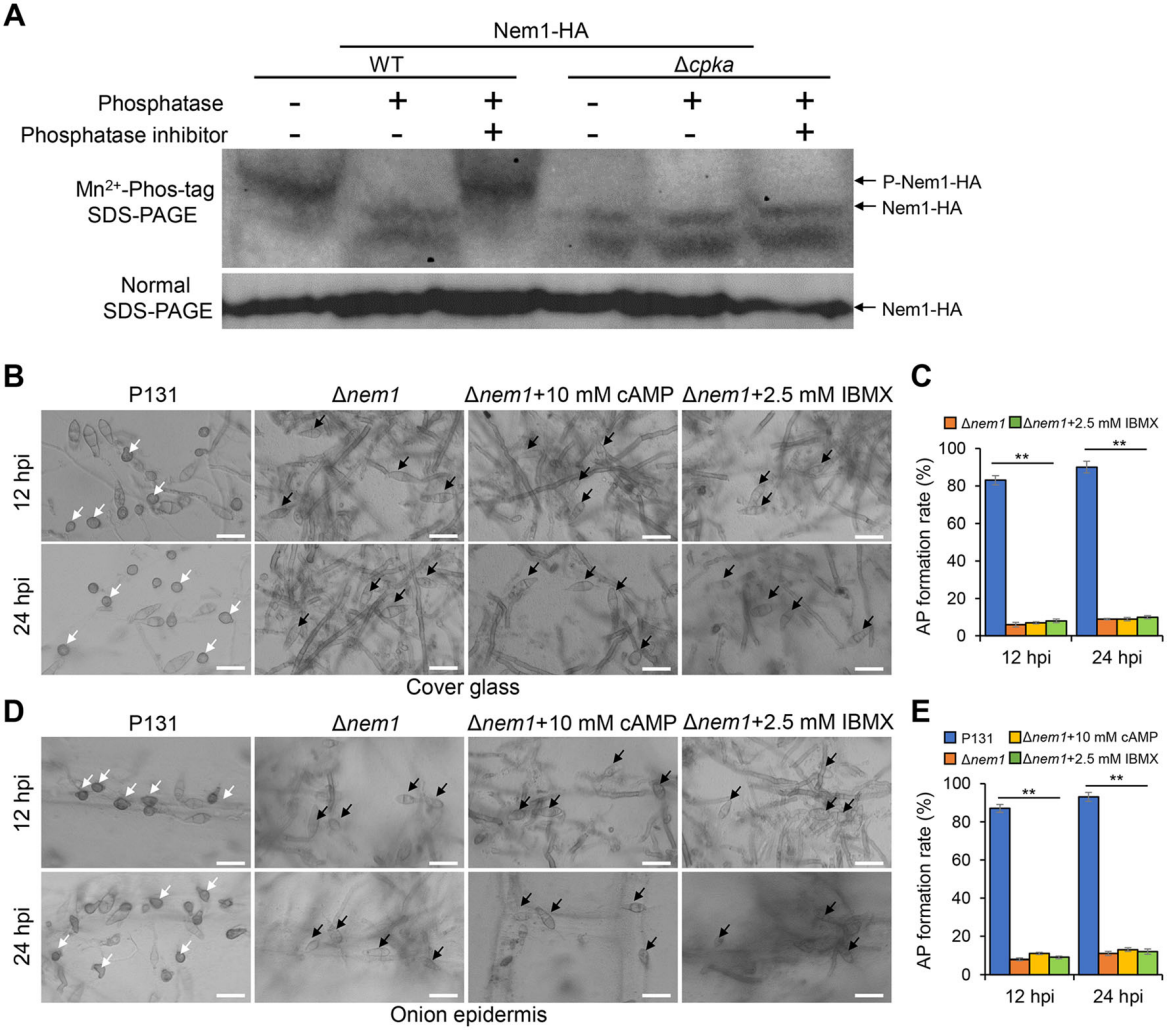
Nem1 is regulated by the cAMP-PKA signaling pathway. **A** Cell extracts of transformants expressed Nem1-HA in WT and Δ*cpka* were subjected to Phos-tag SDS-PAGE. The normal SDS-PAGE was used as a control. **B** Observation of appressoria formation of Δ*nem1* with cAMP or IBMX treatment. Bars, 20 μm. **C** Statistical analysis of appressoria formation rate in **(B)**. Asterisks indicate statistically significant differences (*P* < 0.01). AP, appressoria. **D** Observation of appressoria formation of Δ*nem1* with cAMP or IBMX treatment on the onion epidermis. Scale bar: 20 μm. **E** Statistical analysis of appressoria formation rate in **(D)**. Asterisks indicate statistically significant differences (*P* < 0.01)

Considering that Nem1 is required for appressorium formation in *M. oryzae*, it is possible that Nem1 functions downstream of cAMP-PKA signaling pathway, such as phosphorylated by PKA. To test this possibility, we first deleted *CPKA*, which encodes the catalytic subunit of PKA, using the split-marker approach (Fig. S1A). One mutant termed Δ*cpka* was obtained after PCR verification for further study (Fig. S1C). The Nem1-HA construct was respectively transformed into the wild-type strain and the Δ*cpka* mutant. The resulting transformants WT/Nem1-HA and Δ*cpka*/Nem1-HA were analyzed on gels containing the Phos-tag. Cell extracts of WT/Nem1-HA and Δ*cpka*/Nem1-HA were treated either with the phosphatase or the phosphatase inhibitor, followed by examination of mobility shifts by immunoblotting with the anti-HA antibody. The reduced mobility form of Nem1-HA was present in untreated wild-type cells but not in the phosphatase-treated ones. Nem1-HA of wild-type cells treated with phosphatase inhibitor also exhibited slow mobility. The similar phosphatase-sensitive and slow-moving band was not observed in extracts of untreated Δ*cpka* mutant cells (Fig. 8A), suggesting that CPKA regulates phosphorylation of Nem1.

To further confirm that if appressoria formation of Δ*nem1* was regulated by cAMP-PKA signaling pathway, exogenous 10 mM cAMP or 2.5 mM IBMX were added to observe whether Δ*nem1* recovered in appressoria formation. At 12 hpi and 24 hpi on hydrophobic surface, the appressoria formation rate of Δ*nem1* was around 10%, equivalent to that of Δ*nem1* treated with cAMP or IBMX. While the percentage of the wild-type strain was above 80% (Fig. 8B, C). Similar phenomenon was observed on the onion epidermis (Fig. 8D, E). This result indicates addition of cAMP or IBMX is unable to restore the appressoria formation defect of the Δ*nem1* mutant, consistent with above founding that Nem1 is downstream of the cAMP-PKA signaling pathway.

### Nem1 is phosphorylated by CPKA at Ser303

**Fig. 9:**
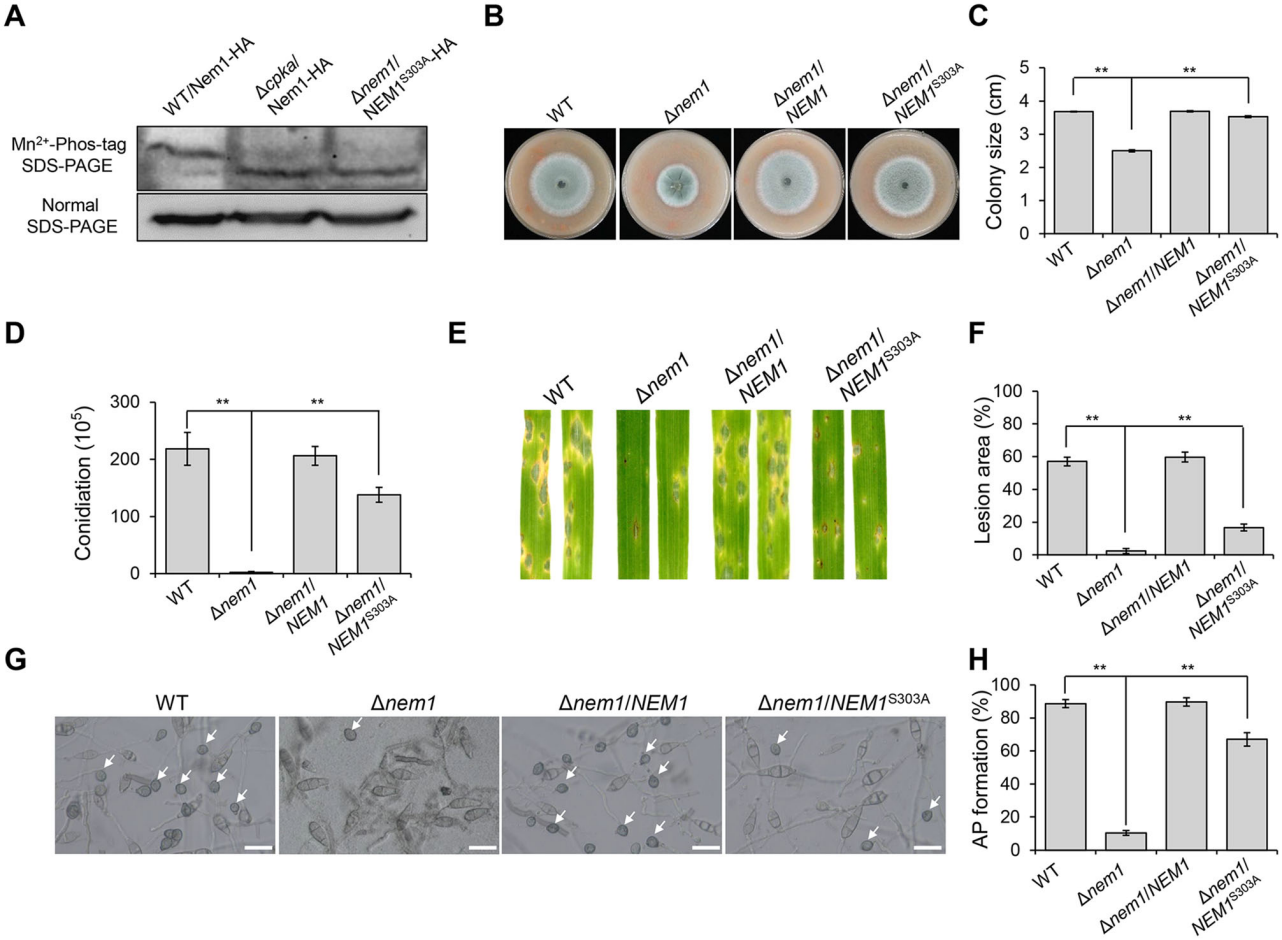
Phosphorylation at Ser303 of Nem1 is important for asexual development and virulence. **A** Cell extracts of the transformant WT/Nem1-HA, Δ*cpka*/Nem1-HA, and Δ*nem1*/NEM1^S303A^-HA were subjected to Phos-tag SDS-PAGE. The normal SDS-PAGE was used as a control. **B** WT, Δ*nem1*, Δ*nem1*/NEM1, and Δ*nem1*/NEM1^S303A^-HA were cultured on OTA plates for 5 days. **C** Statistical analysis of the colony size in **(B)**. Asterisks indicate statistically significant differences (*P* < 0.01). **D** Statistical analysis of conidiation of the indicated strains. Asterisks indicate statistically significant differences (*P* < 0.01). **E** Virulence test of WT, Δ*nem1*, Δ*nem1*/NEM1, and Δ*nem1*/NEM1^S303A^-HA on barley leaves. **F** Statistical analysis of lesion area in **(E)**. Asterisks indicate statistically significant differences (*P* < 0.01). **G** Appressoria formation of WT, Δ*nem1*, Δ*nem1*/NEM1, and Δ*nem1*/NEM1^S303A^-HA on the cover glass at 12 hpi. **H** Statistical analysis of AP formation rates in **(G)**. Asterisks indicate statistically significant differences (*P* < 0.01)

In budding yeast, Nem1 was proved to be phosphorylated at both Ser140 and Ser210. By homologous sequence comparison, Ser303 was speculated to be a candidate phosphorylated site of Nem1 in *M. oryzae*. To confirm this phosphorylation site, the serine was mutated to alanine (S303A). We expressed the Nem1^S303A^-HA in Δ*nem1* and obtained the Δ*nem1*/Nem1^S303A^-HA transformants. We then examined the phosphorylation of Nem1^S303A^-HA using the Phos-tag method. The mobility shift of Nem1^S303A^-HA was similar as Nem1-HA expressed in Δ*cpka*, but faster than Nem1-HA expressed in WT (Fig. 9A). This result showed that Nem1 was phosphorylated at Ser303.

To clarify the biological role of the Nem1 phosphorylation site (Ser303) in *M. oryzae*, phenotypes of Δ*nem1*/NEM1^S303A^-HA were determined. The colony of Δ*nem1*/NEM1^S303A^-HA was significantly smaller than that of WT and the complementary strain Δ*nem1*/NEM1, although obviously greater than that of the Δ*nem1* mutant (Fig. 9B, C). Conidiation of Δ*nem1*/NEM1^S303A^-HA was also obviously recovered to the wild-type level (Fig. 9D). Moreover, the virulence of Δ*nem1*/NEM1^S303A^-HA was evidently recovered comparing with that of the Δ*nem1* mutant (Fig. 9E, F). On hydrophobic surface, the appressorium formation rate of Δ*nem1*/NEM1^S303A^-HA was around 67%, significantly higher than that of the Δ*nem1* mutant (Fig. 9G, H). Altogether, these results indicate that Ser303 of Nem1 plays key roles for development and virulence of the rice blast fungus.

## Discussion

Lipid droplet storages in the fungal conidia have been found to play a key role during appressorium-mediated infection of the plant pathogenic fungi, but the regulatory mechanism of lipid biogenesis and utilization is not clear. In this study, our results showed a crucial role of Nem1 in conidial lipid droplets biogenesis and appressorial lipid droplets’ utilization. These two processes are well coordinated by phosphorylation of Nem1, which is regulated by TOR and cAMP-PKA signaling pathways. Our study provides novel insight into the regulatory mechanism of lipid droplet, as well as appressorium-mediated infection of *M. oryzae*.

As shown in the TLC assay, deletion of *NEM1* altered the lipid profile. Nine different kinds of lipid were isolated and exhibited various sizes, though the overall distribution of the lipid is similar. Lipidomics analysis showed relative content of 11 lipids, among which, PA, PG, PI, DAG, and TAG were reduced in Δ*nem1* compared with that in WT. Because PA tends to synthesize membrane phospholipids in a nutrient-rich condition such as in the CM medium, it is explainable for the reduction of PA although loss of *NEM1* impedes transition from PA to DAG. In a recent study (Zhao et al. 2022), deletion of Pah1 led to obviously increased PA content. This may be caused by blocked transfer of PA to DAG and TAG for lipid storage. The medium used was the nutrient starvation minimum medium, which facilitated PA to synthesize TAG for storage. Notably, both contents of TAG and DAG were reduced in the *NEM1* deletion mutant, which is consistent with the result of lipid droplets staining assay.

In yeast, Pah1 localizes to the nuclear membrane on either side of NVJ (nuclear vacuole junction) and is associated with lipid droplets (Graef 2018; Henne et al. 2019). Phospholipids stored in lipid droplets can be subjected to autophagy by entering the vacuole in case of excess nutrient. Additionally, phospholipids constitute cellular membrane system including the vacuole. The yeast Pah1 mutant was defective in vacuole fusion, which is necessary for autophagy. Moreover, disruption of the *M. oryzae PAH1* led to delayed autophagy. Since Nem1 was localized to lipid droplets and dephosphorylated PAH1 to be functional, thus, we considered that delayed autophagy of Δ*nem1* in this study might resulted from altered lipid composition and defected vacuole fusion. These can be explained by the result that lipid droplets in the Δ*nem1* mutant was delayed in utilization (Fig. 5D).

Nutrient starvation and inactivation of rapamycin complex 1 (TORC1) are considered two ways to promote autophagy. TORC1 is the core component of the TOR signaling pathway regulating processes including lipid, protein, and nucleotide metabolism (Rahman et al. 2018a). TORC1 kinase controls nutrient availability, one way which affects lipid synthesis or storage by directly phosphorylating the Nem1/Spo7–Pah1 axis (Dubots et al. 2014). In budding yeast, nutrition starvation and inactivation of TORC1 protein kinase promote macro-autophagy, a crucial process in cellular activities through degrading cytoplasmic components and organelles in lysosomes/vacuoles (Rahman et al. 2018b; Yin et al. 2020). In our study, autophagy of the Δ*nem1* mutant was blocked upon nitrogen starvation. This finding is consistent with that in *S. cerevisiae*. In *F. graminearum*, rapamycin treatment led to significant increase of LD biogenesis (Liu et al. 2019). In our study, we also proved that rapamycin treatment increases TAG content rather than DAG. This probably because TAG is the main form of lipid stored in LDs and DAG is the intermediate of TAG. This effect was eliminated in the loss of Nem1, indicating Nem1-mediated lipid synthesis is regulated by the TOR signaling.

Disruption of *NEM1* remarkably affected appressorium formation. In *M. oryzae*, several well-studied signaling pathways have been addressed to regulate this important biological process. Among them, the cAMP-PKA signaling pathway regulates the early stage of appressorium formation. In yeast, Nem1 was reported to be phosphorylated by PKA (Su et al. 2018). In this study, we deleted *CPKA*, the catalytic subunit of PKA, and verified CPKA also phosphorylated Nem1. Exogenous addition of cAMP or IBMX was unable to rescue appressorium formation, suggesting Nem1 was downstream of the cAMP-PKA signaling pathway. Lipid utilization was required for appressorium formation (Cai et al. 2020; Chen et al. 2022). Here, we provided a novel regulatory mechanism of the cAMP-PKA signaling pathway regulated lipid utilization and appressorium formation.

Phos-tag SDS-PAGE analysis shows Cpka phosphorylates NEM1 at Ser303. The site mutant S303A was generated to study the biological function of this phosphorylation site. Pleiotropic defects including vegetative growth, conidiation, appressorium formation, and virulence of the site mutant were all partially restored. This probably results from more than one residue phosphorylated by CPKA since the Ser303 serine was found through homologous sequence alignment with *S. cerevisiae*, in which Ser140 and Ser210 were both phosphorylated to be functional. A more suitable phosphorylation site confirmation method should be used, such as mass spectrometry analysis.

In conclusion, this study has demonstrated that Nem1, a lipid metabolism-related gene, is important for asexual development, appressorium formation, and pathogenicity. We proposed a model to understand the Nem1-mediated regulatory mechanism in *M. oryzae* (Fig. S5). In this model, Nem1 is a de-phosphorylated pattern in the conidium (or in the hypha), which is important for lipid droplets biogenesis and accumulation under the nutrient-rich condition. While Nem1 can be phosphorylated at Ser303 by cAMP-PKA signaling pathway during the appressorium formation stage. Phosphorylated Nem1 may be functional to facilitate the lipid droplets degradation through the autophagy process or enzymatic degradation. Upon nutrient starvation or TORC1 inactivation, phosphorylation of Nem1 can be inhibited by responding to the TOR signaling pathway, leading to prevention of the appressorium formation process. A similar regulatory mechanism can be also found in perilipin LDP1, another lipid droplet-associated protein in *M. oryzae* (Cai et al. 2020). Therefore, Nem1-mediated regulatory mechanism may be helpful for developing fungicide in future.

## Materials and methods

### Strains and culture conditions

The strain P131 was used as the *M. oryzae* wild-type (Xue et al. 2012). All strains listed in Table S1 were cultured on OTA plates at 28 °C in the incubator. Colony diameters on the OTA plates were measured at 5 days post-inoculation (dpi). Conidia from 7-day-old colonies cultured on OTA plates were harvested for testing. Strains cultured in liquid complete medium (CM) at 28 °C were used for DNA and RNA extraction.

To test fungal response to environmental stresses, mycelial blocks were inoculated onto the CM plates supplemented with 0.2 mg/mL Congo red (Sigma-Aldrich, St. Louis, MO, USA), 0.1 mg/mL CFW (Sigma-Aldrich, St. Louis, MO, USA), 0.005% SDS, 0.7 M NaCl, 1.0 M sorbitol, 5–20 mM H_2_O_2_, 6.25–500 nM rapamycin (53,123–88-9, Merck, Darmstadt, Germany), respectively. The colony diameters were measured at 5 dpi.

### Phylogenetic analysis

Nem1 protein sequences in various species including *M.*, *Colletotrichum truncatum*, *V. dahlia*, *F. oxysporum*, *G. tritici*, *Neurospora crassa*, *Aspergillus melleus*, *Schizosaccharomyces pombe*, *S. cerevisiae*, and *Candida albicans* were downloaded from the NCBI database. The neighbor-joining phylogenetic tree was constructed using MEGA 7.0 in the p-distance model with 1000 bootstrap replicates.

### Quantitative real-time PCR analysis

To evaluate the expression profile of *NEM1*, conidia produced from OTA plates, appressoria (3 hpi and 12 hpi) harvested from Teflon films (0.2 mm thickness), and invasive hyphae (18, 24, and 48 hpi) collected from inoculated epidermis of barley leaves were harvested to extract total RNA for preparing cDNA templates. The qRT-PCR was performed using an SYBR Green PCR Master Mix kit (Takara, Dalian, China) on an ABI 7500 real-time PCR system (Applied Biosystems, Foster City, CA, USA). The *M. oryzae* *GAPDH* was used as the reference gene.

### Gene deletion and complementation

The split-PCR strategy was used for gene disruption as previously described (Goswami 2012; Shi et al. 2022). For deleting *NEM1* or *CPKA*, hygromycin (*HYG*) was used as a bridge gene to generate recombinant fragments. PEG-mediated genetic transformation was adopted, and transformants were screened by 250 μg/mL hygromycin B (Roche Diagnostics, Indianapolis, IN, USA). Left boarder (LB) and right boarder (RB) of *NEM1* or *CPKA* from transformants were confirmed by PCR using the LBCK/ HPT-up and RBCK/HPT-down primer pairs (Table S3). Gene deletion was further verified by PCR of an internal fragment of *NEM1* or *CPKA*. For obtaining complementary strains, a vector containing 1.5 kb native promoter region, the *NEM1* or *CPKA* gene coding region and the adjacent 0.5 kb downstream region were separately transferred into the Δ*nem1* and Δ*cpka* mutant. The CM plates supplemented with 400 μg/mL G418 were used to select the complementary transformants followed by PCR and phenotype verification.

### Observation of conidiophores and conidia

Mycelia of P131, *NEM1* deletion mutants and the complementary strain grown on OTA media for 5 days were scraped to a sterilized tube added with 1 mL of sterilized ddH_2_O in the tube. The mycelia were broken with a vibrator for 30 s and transferred to a new thicker OTA plate, and evenly smeared on the surface of the medium. The mixture was dried and cultured at 28 °C for 36 h till new hyphae grew on the surface. The hyphae were stirred and washed with sterile water, and then dried. The sterilized blade was used to cut the long piece of mycelium at the edge of the colony, put it on the slide with the front facing up and keep moisture, followed by incubated it in the 28 °C incubator under light, and took photos under the microscope after 18 h.

### Appressorium formation

Conidial suspension drops (2 × 10^5^ spores/mL) were inoculated on the microscope cover glass (12540A, ThermoFisher, Pittsburgh, PA, USA) or onion epidermis and incubated at 28 °C under darkness for 12 h and 24 h before observation. Appressorium formation rates were counted as the number of appressoria produced in every 100 conidia. By evaluating effect of cAMP and IBMX on appressorium formation, 10 mM cAMP and 2.5 mM IBMX were separately added to the conidial suspension drops followed by inoculated on the cover glass or onion epidermis.

### Staining assays

The conidia of different strains were collected from OTA plates and stained with 10 μg/mL CFW solution (Sigma-Aldrich, St. Louis, MO, USA). The stained conidia were observed and photographed under a fluorescence microscope (Ni90 microscope, Nikon, Tokyo, Japan). The proportion of conidia with different number of septa was counted. The hyphae grew on the coverslip, which was previously inserted to the colony edge on the OTA plate. Hyphae tips on the coverslip were stained with CFW solution for 5 min. The samples were washed twice with PBS buffer before observed and photographed under the fluorescence microscope (Ni90).

The ROS staining assay was performed as previously described (Chen et al. 2014). Barley leaves infected by the indicated strains at 30 hpi were stained with 1 mg/mL DAB (Sigma-Aldrich) solution (pH 3.8) for 12 h under darkness, followed by de-stained with ethanol/acetic acid solution (v/v, 94:4) for 24 h on the shaker with de-stained buffer changing. 0.5 μM diphenyleneiodonium (DPI) was added onto the conidial droplets to eliminate ROS. The whole leaf was observed and photographed under the fluorescence microscope (Ni90).

For lipid droplet staining assay, vegetative hyphae and conidia were collected in a 2 mL centrifuge tube and incubated with 2 μM BODIPY 493/503 (Sigma-Aldrich) solution in PBS in the dark by shaking gently for 5 min at the room temperature. Samples were then transferred to the cover slide and washed with a quick rinse using PBS before observation under a confocal microscope (TCS SP8; Leica). The maximum wavelength of excitation/emission is 493/503 nm. Appressoria were stained on the hydrophobic slide with 2 μM BODIPY 493/503 adding on the conidial suspension drop cultured for 8 h.

### Virulence test assays

Rice seedlings (*Oryza sativa* cv. LTH) grown for four weeks and the barley (cv. E9) grown for one week were used for virulence tests. For spraying inoculation, conidial concentration was adjusted to 3 × 10^4^ conidia/mL in 0.025% Tween 20 to spray barley and 1.5 × 10^5^ to spray rice leaves. The leaves were incubated under a full humidity condition at 28 °C with 12 h of darkness and 12 h of light successively in a day. The inoculated leaves were observed and photographed 4 days later. For infection process observation, conidia suspension (2 × 10^5^) drops were inoculated onto barley leaves. The leaf epidermis was collected and observed at 24, 30, and 36 hpi under the fluorescence microscope (Ni90).

### Subcellular localization observation

The gene coding region of *NEM1* linked with the native promoter was amplified and ligated into the *N*-terminus of the GFP gene in the vector pGTN (Table S1; Table S2). The subsequent vector pGTN-NEM1 was transformed into Δ*nem1*. Transformants at different developmental and infection stages were used to observe subcellular localization under the confocal laser scanning microscope (TCS SP8; Leica).

### Autophagy analysis

Autophagy analysis was conducted as previously described (Chen et al. 2022; Ren et al. 2022; Yin et al. 2020). The construct of MoAtg8 fused with GFP at its *N*-terminus was transformed into the WT and Δ*nem1* mutant. Transformants were screened by Western blotting, followed by cultured in CM for 48 h. Mycelia were rinsed with water and transferred to the MM with nitrogen starvation (MM-N) for 2 h and 6 h to induce nonselective autophagy. The fluorescence signals were observed under a confocal microscope (TCS SP8; Leica). Proteins were extracted and analyzed by Western blotting with the anti-GFP antibody. The amount of free GFP and GFP-Atg8 was quantified by densitometric analysis with the ImageJ software.

### Lipid analysis

Lipid was extracted as previously described (Bligh et al. 1959; Khoomrung et al. 2013). Mycelia were harvested from liquid CM cultured at 28 °C with shaking (160 rpm) for 48 h and freeze-dried. The samples were further incubated in hot isopropanol (75 °C) containing 0.05% (v/v) butylated hydroxytoluene (Sigma-Aldrich) for 15 min. Total lipid was extracted using chloroform/methanol (2:1, v/v) added with 0.01% butylated hydroxytoluene. This step was repeated five times with shaking. Then 1.0 M KCl and ddH_2_O were added to the sample for centrifugation, and the upper phase was discarded. The solvent was evaporated under a nitrogen gas stream and re-dissolved in chloroform (5 mg/mL) before detecting by TLC and lipidomics analysis.

After loading total lipid extracted from the samples, TLC plates were incubated at 110 °C for 90 min and samples were spotted at one corner of the plates. Chloroform/ethanol/ammonium hydroxide (65:25:2, v/v/v) and chloroform/ethanol/acetic acid/water (85:15:10:3, v/v/v/v) were used for the first and second dimensional separation, respectively. After drying, the plates were exposed to iodine vapor for 90 s in the tank. The lipid contents were measured as described using an Agilent HPLC system coupled with a triple quadrupole/ion trap 4000 QTrap mass spectrometer (Applied Biosystems) (Nakamura et al. 2014; Zhao et al. 2022).

### Phos-tag analysis

Phos-tag assays were performed as previously described (Li et al. 2017). The Nem1-HA fusion construct was transferred into the wild-type strain and the Δ*nem1* mutant. The positive transformants were cultured in liquid CM for 48 h. For protein isolation, 200 mg of mycelia was ground into powder in liquid nitrogen and re-suspended in 1 mL of extraction buffer (10 mM Tris–HCl [pH 7.5], 150 mM NaCl, 0.5 mM EDTA, 0.5% NP40) to which 1 mM PMSF, 10 μL of protease inhibitor cocktail (Sigma), and 10 μL of phosphatase inhibitor cocktail 3 (Sigma) had to be freshly added. For the preparation of the phosphatase-treated cell lysates, the phosphatase inhibitor cocktail was omitted for 2.5 U/mL alkaline phosphatase (P6774; Sigma), and the sample was incubated for 1 h with the addition of 1 mM MgCl_2_ (37°C). The samples were further resolved on 8% SDS–polyacrylamide gels prepared with 50 μM acrylamide-pendant Phos-tag ligand and 100 μM MnCl_2_ according to the instructions provided by the Phos-tag consortium. Gels were electrophoresed at 60 V/gel for 5 h. Prior to transfer, gels were first equilibrated in transfer buffer containing 5 mM EDTA for 20 min three times and then in transfer buffer without EDTA for 10 min by shaking. Protein transfer from the Mn^2+^-phos-tag™ acrylamide gel to the PVDF membrane was performed overnight at 80 V at 4 °C, and then the membrane was analyzed by Western blotting using anti-HA antibodies.

### Statistical analysis

All values represent the mean of at least three biological replicates. Error bars indicate the standard deviation. Statistical comparisons were performed using one-way analysis of variance (ANOVA) (*P* < 0.05) in SPSS 19.0 software (IBM, New York, USA).

## Supplementary Information

Below is the link to the electronic supplementary material.

Supplementary file1 (DOCX 2875 KB)

## Data Availability

The data that support the findings of this study are available from the corresponding author upon reasonable request.
