# Supplementary material for: A lipid droplet-associated protein Nem1 regulates appressorium function for infection of Magnaporthe oryzae
Source: aBIOTECH. 2023 Feb 18;4(2):108–23. doi: 10.1007/s42994-023-00098-5 (PMC10423190; doi:10.1007/s42994-023-00098-5)
Supplement: Supplementary file 1 — Supplementary file1 (DOCX 2875 KB) [file 42994_2023_98_MOESM1_ESM.docx]

**Supplemental data:** **Chen *et al.* (2022).** **A lipid droplet associated protein Nem1 regulates appressorium function for infection of *Magnaporthe oryzae***

**This Supplemental data includes:**

**Supplemental Figure S1 to S5**

**Supplemental Table S1 to S3**


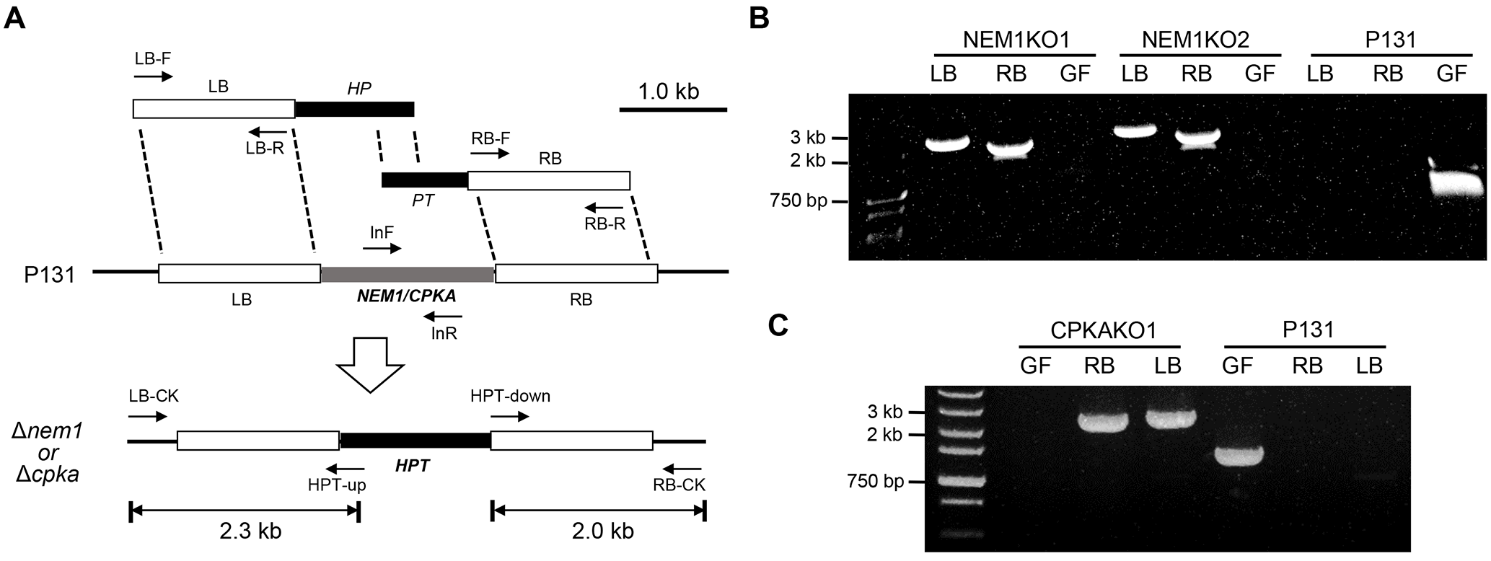


**Fig. S1.** Deletion of *NEM1* and *CPKA*. **A** A schematic diagram of *NEM1* and *CPKA* deletion. **B** PCR confirmation of two *NEM1* deletion mutants. **C** PCR confirmation of the *CPKA* deletion mutant.


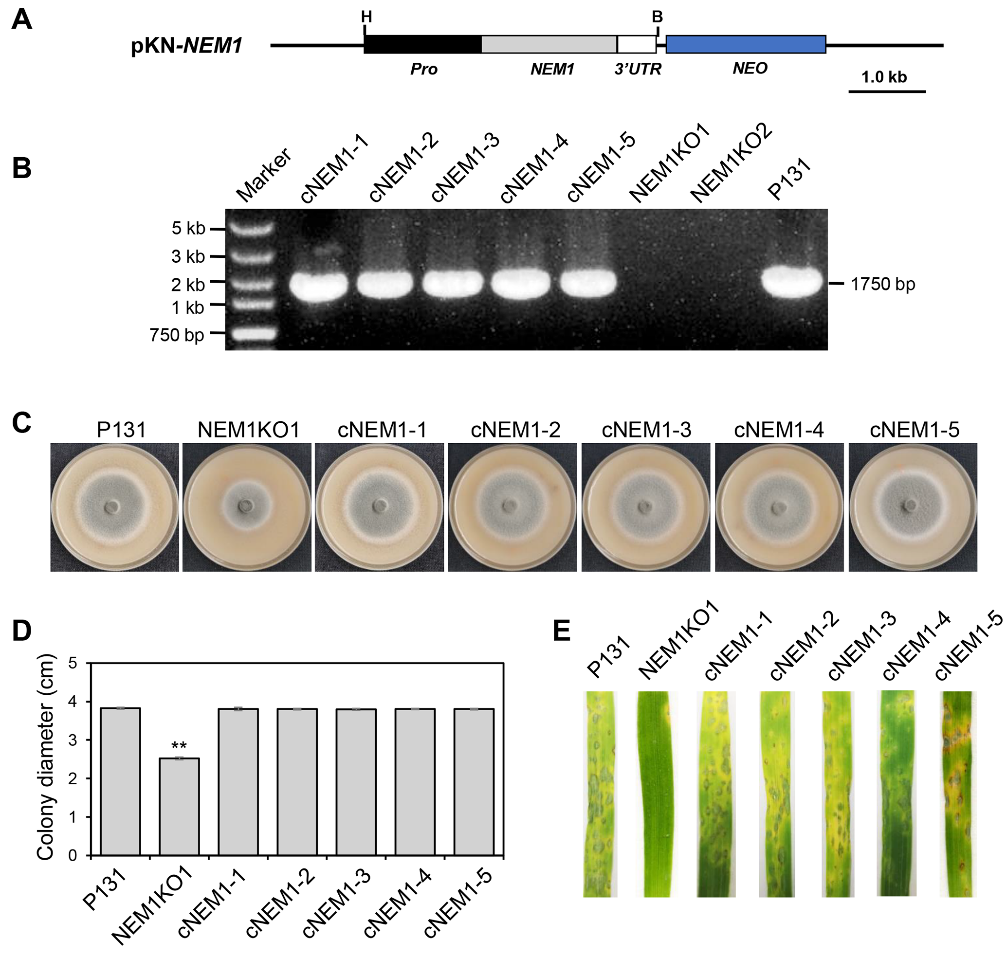


**Fig. S2.** Complementation of the *NEM1* deletion mutants. **A** A schematic diagram of construction of the complementary vector. **B** Verification of complementary strains by amplification of the NEM1 gene using PCR. **C** Colony growth of the complementary strains. **D** Statistical analysis of the colony diameters in (C). Asterisks indicate statistically significant differences (*P* < 0.01). **E** Virulence test of the complementary strains on host barley leaves.


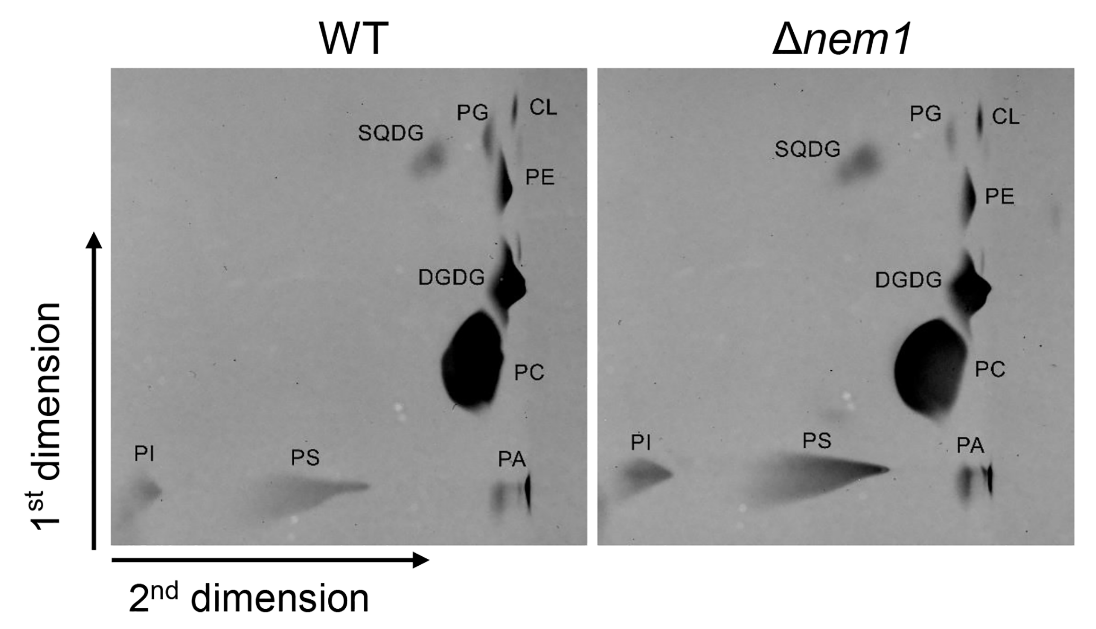


**Fig. S3.** Thin-layer chromatography (TLC) analysis of lipid composition of WT and Δ*nem1*. Spots were indicated with black abbreviations of the corresponding lipid. CL, cardiolipin; DGDG, digalactosyl diglyceride; SQDG, sulphoquinovosyldiglyceride.


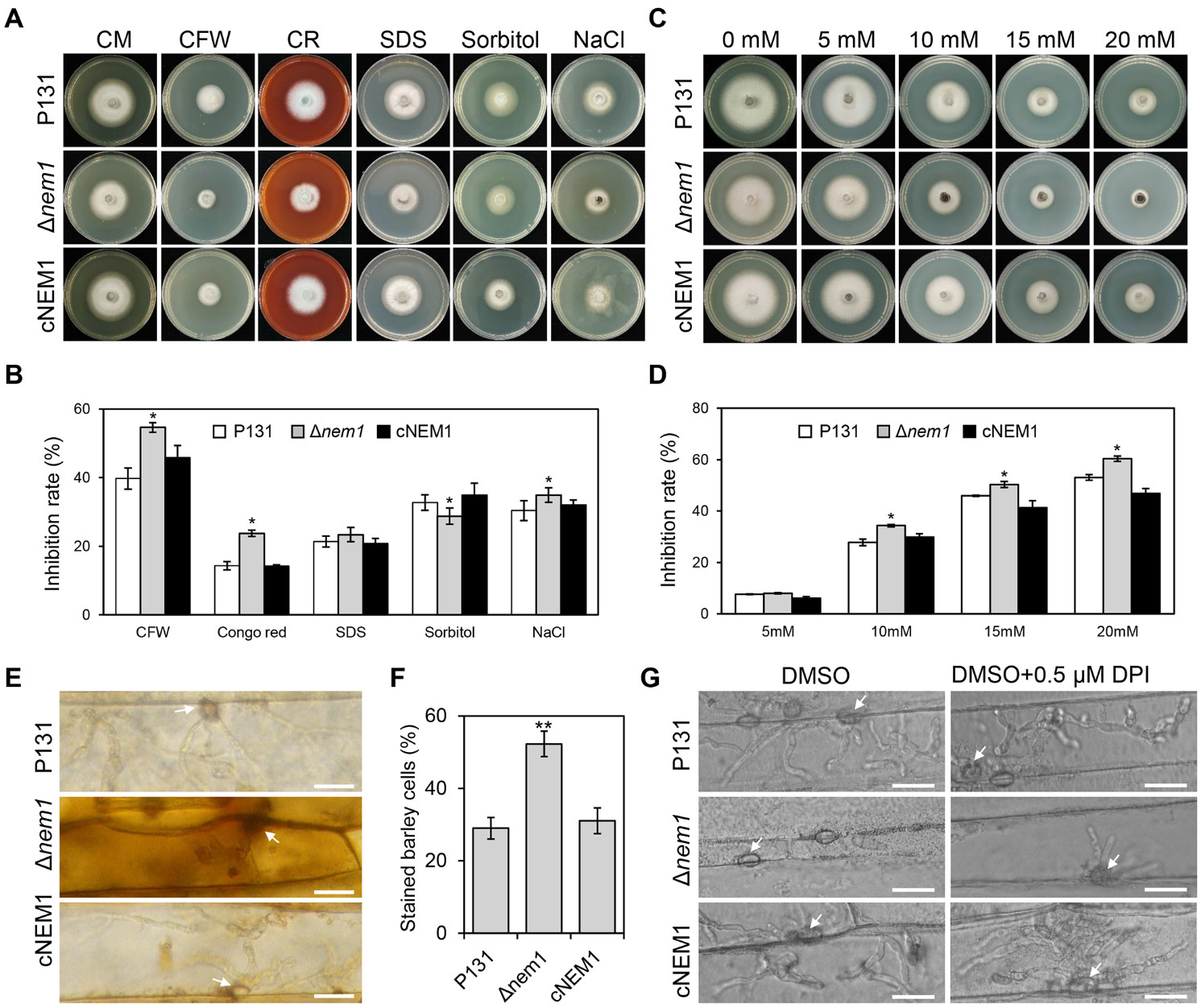


**Fig. S4.** NEM1 is involved in cell wall integrity and protecting against host defense response. **A** P131, Δ*nem1*, and cNEM1 were cultured on CM plates added with CFW, CR, SDS, sorbitol, and NaCl for 5 days. **B** Statistical analysis of growth inhibition rate of CFW, Congo red, SDS, sorbitol, and NaCl (*P* < 0.05). **C** P131, Δ*nem1*, and cNEM1 were cultured on CM plates added with a gradient concentration of H_2_O_2_ from 5 mM to 20 mM. **D** Statistical analysis of growth inhibition rate of different concentration of H_2_O_2_ (*P* < 0.05). **E** Host cellular ROS stained by DAB in different strains infected barley cells at 30 hpi. White arrows indicate appressoria. Scale bar: 20 μm. **F** Statistical analysis of percentage of stained barley cells (*P* < 0.01). **G** Observation of invasive growth at 30 hpi when treated with diphenyleneiodonium (DPI). Dimethyl sulphoxide (DMSO) treatment was used as a control. Scale bar: 20 μm.


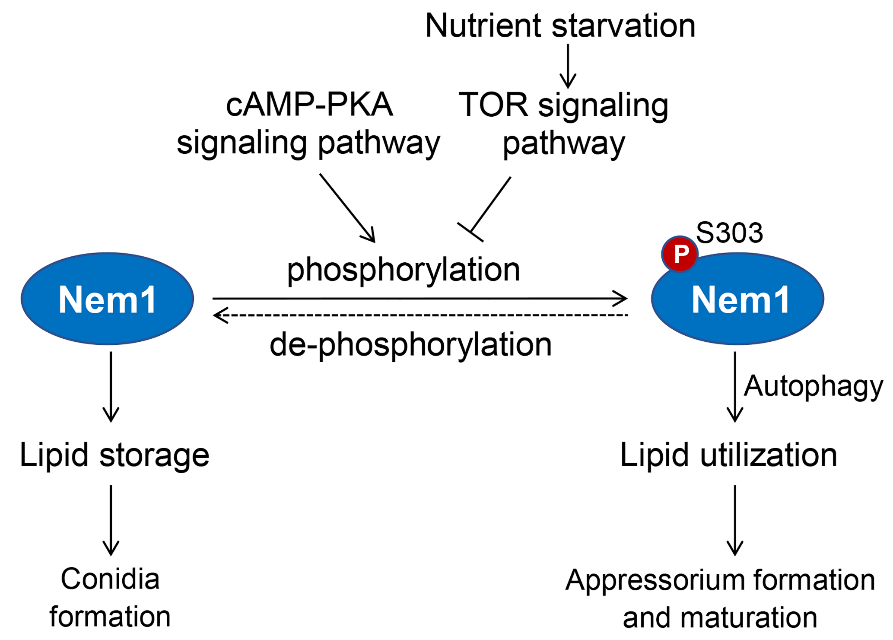


**Fig. S5.** A proposed model for the potential regulatory mechanism of Nem1 in *M. oryzae*. Nem1 is a de-phosphorylated pattern in the conidium, which is important for lipid droplets biogenesis and accumulation under the nutrient-rich condition. Nem1 can be phosphorylated at ser-303 through the cAMP-PKA signaling pathway during the appressorium formation stage. Phosphorylated Nem1 facilitates lipid droplets utilization by the autophagy process or enzymatic degradation. Upon nutrient starvation or TORC1 inactivation, phosphorylation of Nem1 can be inhibited by responding to the TOR signaling pathway, leading to block of the appressoria formation process.

**Table S1 Fungal strains used in this study.**

| **Strains** | **Discriptions** | **References** |
| --- | --- | --- |
| P131  GFP-Nem1  NEM1KO1  NEM1KO2  cNEM1  Δ*nem1*  WT/Atg8-GFP  Δ*nem1*/Atg8-GFP  Δ*cpka*  cCPKA  WT/Nem1-HA  Δ*cpka*/Nem1-HA  cCPKA/NEM1-HA  Δ*nem1*/*NEM1*  Δ*nem1*/*NEM1*^S303A^ | A wild-type isolate of *M. oryzae*  The localization strain of NEM1  The first *NEM1* deletion mutants of P131  The second *NEM1* deletion mutants of P131  The complementary strain of *NEM1* deletion mutants  NEM1KO1, the selected *NEM1* deletion mutant for further experiments  The transformant expresses Atg8-GFP fused protein in P131  The transformant expresses Atg8-GFP fused protein in Δ*nem1*  The *CPKA* deletion mutant  The complementary strain of the *CPKA* deletion mutant  The transformant expresses Nem1-HA in P131  The transformant expresses Nem1-HA in Δ*cpka*  The transformant expresses Nem1-HA in cCPKA  The transformant expresses Nem1 mutated vector Nem1 ^S303A^ -HA in Δ*nem1* | (Xue et al, 2012)  This study  This study  This study  This study  This study  This study  This study  This study  This study  This study  This study  This study  This study |
|  |  |  |

**Table S2 Plasmids used in this study.**

| **Names** | **Descriptions** |
| --- | --- |
| pKN | The plasmid used to construct complementary vectors, with the *NPTII* gene as a selective marker inserted into pKS^+^ (Yang et al., 2010). |
| pKN-*NEM1*  pKN-*CPKA*  pGTN | The *NEM1* complementary vector; *NEM1* gene containing 1.5 kb promoter and 3’UTR were amplified and inserted into pKN.  The *CPKA* complementary vector; *CPKA* gene containing 1.5 kb promoter and 0.5 kb 3’UTR were amplified and inserted into pKN.  The plasmid used to construct vectors to express target genes (Yang et al., 2010) |
| pGTN-*NEM1*  pGTN-*ATG8*  pYIP102  pYIP102-*NEM1* | The plasmid for subcellular localization of NEM1; 1.5 kb promoter and the coding region of *NEM1* was cloned into the vector pGTN.  The plasmid for subcellular localization of ATG8; 1.5 kb promoter and the coding region of *ATG8* was cloned into the vector pGTN.  The plasmid used to construct vectors to express target genes and detect with Western blot  The plasmid used to express NEM1 tagged with tandem HA and Flag; 1.5 kb native promoter and coding region of *NEM1* was cloned into vector pYIP102. |
| pYIP102-*NEM1*^S303A^ | The plasmid used to express Ser-303 mutated NEM1 tagged with tandem HA and Flag; 1.5 kb native promoter and coding region of *NEM1* was cloned into vector pYIP102. |

**Table S3** **Primers used in this study.**

| **Primers** | **Sequences (5'–3')** |
| --- | --- |
| HPT-up | GACAGACGTCGCGGTGAGTT |
| HPT-down | TCTGGACCGATGGCTGTGTAG |
| HPT-F1 | CTCCGACCTGATGCAGCTCT |
| HPT-R1 | CTCGCTCCAGTCAATGACC |
| HPT-F | CTTGGCTGGAGCTAGTGGAGGT |
| HPT-R | CCCGGTCGGCATCTACTCTATTC |
| NEM1LB-CK | CACATTCGTTGCATCGTC |
| NEM1LB-F | CACCATGCGCCTCTGTGC |
| NEM1LB-R | ACCTCCACTAGCTCCAGCCAAGTCCGTTCGACCGGTCTAG |
| NEM1RB-F | GAATAGAGTAGATGCCGACCGGGTGGCATGCACGTGATCGT |
| NEM1RB-R | AGGTCTGGAACAGAGAAC |
| NEM1RB-CK | GACGCTGACACCGACGGT |
| NST1InF  NST1InR  CPKALB-CK  CPKALB-F  CPKALR  CPKARB-F  CPKARB-R  CPKARB-CK  CPKAInF  CPKAInR  NEM1KN-F | CGAATCAACGCCGCTATT  GACTGGATCCGCGTACTC  GTTAGTTCCATTGCATAC  CAAGTTCAGAATCGGTCT  ACCTCCACTAGCTCCAGCCAAGACAGAACTGTCCGAATC  GAATAGAGTAGATGCCGACCGGGTCATGCTGTGACGCACT  TACAGATGGGCTTGATTA  CAGAAAGGCGAGTTAGAT  CTCGGCCAACTTCCAAC  TATGGTGCATCTATGTCC  GGTATCGATAAGCTTTTCCCACCATGCGCCTCT |
| Nem1KN-R | AATCTAGAACTAGTGGATCCGTAGAATTCATGCCACAG |
| Nem1GTN-F | TCGAGGTCGACGGTATCGATAAGCTTTTCCCACCATGCGCCTCT |
| Nem1GTN-R | CCAGCACCTCTAGAACTAGTGGATCCTGCCATATGTTGGCCGT |
| NEM1-yip102F | CATCTCGAGCACCATGCGCCTCTGTG |
| NEM1-yip102R | TATCCCGGGTGCCATATGTTGGCCGT |
| M303F | CCAGCTCAAAGCCCCTACGTCGCCTG |
| M303R | GACGTAGGGGCTTTGAGCTGGGCGGA |
| ATG8GTN-F | TCGAGGTCGACGGTATCGATAAGCTTAGACAGCTGGACGTGGTC |
| ATG8GTN-R  NEM1-qF | CCAGCACCTCTAGAACTAGTGGATCCCTCGACTTCCTCAAACAG  TTGAGTCAGAGAGGAAGT |
| NEM1-qR | AGAAGTGCTCGGACGTCG |
| GAPDH-qF  GAPDH-qR | TCTTCACCACCACCGACAAG  CGACTTCTCGTTGACACCCA |
